# Global utilization of low-dose corticosteroids in severe sepsis and septic shock: a report from the PROGRESS registry

**DOI:** 10.1186/cc9044

**Published:** 2010-06-03

**Authors:** Richard Beale, Jonathan M Janes, Frank M Brunkhorst, Geoffrey Dobb, Mitchell M Levy, Greg S Martin, Graham Ramsay, Eliezer Silva, Charles L Sprung, Benoit Vallet, Jean-Louis Vincent, Timothy M Costigan, Amy G Leishman, Mark D Williams, Konrad Reinhart

**Affiliations:** 1 Division of Asthma, Allergy and Lung Biology, King's College London, Guy's, Campus, Great Maze Pond, London, SE1 9RT, UK; 2 Intensive Care Unit, Guy's and St. Thomas' NHS Foundation Trust, St. Thomas' Hospital, Westminster Bridge Road, London, SE1 7EH, UK; 3 Lilly Research Laboratories, Eli Lilly and Company, Lilly Corporate Center, Indianapolis, IN 46285, USA; 4 Department of Anesthesiology and Intensive Care, Friedrich-Schiller University, Erlanger Allee 101, Jena, 07743, Germany; 5 Royal Perth Hospital, Wellington Street, Perth, WA, Australia; 6 Medical Intensive Care Unit, Rhode Island Hospital, 593 Eddy Street, MICU Main 7, Providence, RI 02903, USA; 7 Division of Pulmonary, Allergy and Critical Care, Department of Medicine, Emory University, 49 Jesse Hill Jr Drive S. E., Atlanta, GA 30303, USA; 8 Mid Essex Hospital Services NHS Trust, Broomfield Hospital, Court Road, Broomfield, Chelmsford, CM1 7WE, UK; 9 Intensive Care Unit, Hospital Israelita Albert Einstein, Avenida Albert Einstein 627, Sao Paulo, 05651-901, Brazil; 10 Department of Anesthesiology and Critical Care Medicine, Hadassah Hebrew University Medical Center, Ein-Karem, Jerusalem, Israel; 11 Department of Anesthesiology and Intensive Care, University Hospital of Lille, Univ Lille Nord de France, F-590000, France; 12 Department of Intensive Care, Erasme University Hospital, Université Libre de Bruxelles, Route de Lennik 808, 1070, Brussels, Belgium

## Abstract

**Introduction:**

The benefits and use of low-dose corticosteroids (LDCs) in severe sepsis and septic shock remain controversial. Surviving sepsis campaign guidelines suggest LDC use for septic shock patients poorly responsive to fluid resuscitation and vasopressor therapy. Their use is suspected to be wide-spread, but paucity of data regarding global practice exists. The purpose of this study was to compare baseline characteristics and clinical outcomes of patients treated or not treated with LDC from the international PROGRESS (PROmoting Global Research Excellence in Severe Sepsis) cohort study of severe sepsis.

**Methods:**

Patients enrolled in the PROGRESS registry were evaluated for use of vasopressor and LDC (equivalent or lesser potency to hydrocortisone 50 mg six-hourly plus 50 μg 9-alpha-fludrocortisone) for treatment of severe sepsis at any time in intensive care units (ICUs). Baseline characteristics and hospital mortality were analyzed, and logistic regression techniques used to develop propensity score and outcome models adjusted for baseline imbalances between groups.

**Results:**

A total of 8,968 patients with severe sepsis and sufficient data for analysis were studied. A total of 79.8% (7,160/8,968) of patients received vasopressors, and 34.0% (3,051/8,968) of patients received LDC. Regional use of LDC was highest in Europe (51.1%) and lowest in Asia (21.6%). Country use was highest in Brazil (62.9%) and lowest in Malaysia (9.0%). A total of 14.2% of patients on LDC were not receiving any vasopressor therapy. LDC patients were older, had more co-morbidities and higher disease severity scores. Patients receiving LDC spent longer in ICU than patients who did not (median of 12 versus 8 days; *P *<0.001). Overall hospital mortality rates were greater in the LDC than in the non-LDC group (58.0% versus 43.0%; *P *<0.001). After adjusting for baseline imbalances, in all mortality models (with vasopressor use), a consistent association remained between LDC and hospital mortality (odds ratios varying from 1.30 to 1.47).

**Conclusions:**

Widespread use of LDC for the treatment of severe sepsis with significant regional and country variation exists. In this study, 14.2% of patients received LDC despite the absence of evidence of shock. Hospital mortality was higher in the LDC group and remained higher after adjustment for key determinates of mortality.

## Introduction

Debate regarding the utility of corticosteroids in the treatment of severe sepsis and septic shock has continued over many years [1-3]. Much of the debate has related to the characterization of the patient population that is most likely to benefit from treatment, optimum dose, and duration of treatment. Although it is now generally accepted that short courses of high-dose corticosteroids do not decrease mortality from severe sepsis and septic shock [4-6], longer courses of low-dose corticosteroids (LDC) have been shown to improve systemic hemodynamics and reduce the time on vasopressor treatment [2,7]. Following the French multi-center study demonstrating that low-dose corticosteroids reduced mortality in patients with septic shock and relative adrenal insufficiency refractory to vasopressor treatment [8], the use of low-dose corticosteroids was incorporated into the 2004 Surviving Sepsis Campaign guidelines [9], recommending their use for patients with septic shock who require vasopressor treatment despite adequate fluid resuscitation. Importantly, they were not recommended for sepsis in the absence of shock. Subsequently, it is believed that the use of low-dose corticosteroids in clinical practice increased. Questions, however, were raised as to the applicability of these results to the wider intensive care unit (ICU) population as well as concerns as to the suitability of more widespread use of low-dose corticosteroids in severe sepsis [10-12]. A retrospective case-control study from a single US site with 10,285 patients [13] reported that 26% of critically ill patients admitted to the ICU were treated with steroids. After adjustment for baseline differences in disease severity and co-morbidities, these patients experienced a higher mortality and morbidity compared to controls that did not receive corticosteroids. The CORTICUS study of corticosteroids in patients with septic shock reported that low-dose corticosteroids treatment was not associated with a mortality reduction in the overall population or those with *relative adrenal insufficiency *(critical illness-related corticosteroid insufficiency) [14]. The overall mortality rate in the trial, however, was lower than in the French study [8]. The differing results in the *relative adrenal insufficiency *subgroups between the French and CORTICUS studies [8,14] resulted in new recommendations for steroid use in a more recent Surviving Sepsis Campaign guidelines consensus statement [15]. The recommendations suggest use only in adult patients in septic shock who are poorly responsive to fluid resuscitation and vasopressor therapy, but again, not for patients with sepsis in the absence of shock. A meta-analysis of randomized trial results of corticosteroids in the treatment of severe sepsis and septic shock [16] suggested that the administration of low-dose corticosteroids for at least five days had a beneficial effect on short-term mortality. Other recent meta-analyses [17,18] evaluating the effects of corticosteroids for the treatment of septic shock, found more heterogeneous effects on mortality, but suggested that low-dose corticosteroids significantly reduce the incidence of vasopressor-dependent shock [18] and improve shock reversal [17]. In contrast to the Annane *et al*. 2009 meta-analysis results [16], a recent observational study [19] found no association between the administration of low-dose corticosteroids in septic shock and reduction of mortality, results echoed in a Bayesian analysis of pivotal trials in severe sepsis [20]. Thus it can be seen that the potential benefits and use of low-dose corticosteroids in severe sepsis and septic shock remains controversial. Although the use of low-dose corticosteroids for severe sepsis is suspected to be wide-spread, there is paucity of data regarding global practice.

The global PROGRESS (PROmoting Global Research Excellence in Severe Sepsis) registry was developed and designed to provide a description of the management and outcomes of severe sepsis in intensive care units, reflecting everyday clinical practice [21]. Although the PROGRESS registry was not specifically designed to assess the use of low-dose corticosteroids, their use was one of a number of therapeutic interventions on which data were collected. The purpose of this sub-study is to describe the use of low-dose corticosteroids in severe sepsis across ICUs globally and compare baseline characteristics and outcomes in treated and non-treated patients. Some results relating to steroid use in severe sepsis from the PROGRESS registry were reported in the form of an abstract at the Society of Critical Care Medicine (SCCM) in 2006 [22].

## Materials and methods

### Study design

PROGRESS was an international, non-interventional, multi-center, prospective, observational study of all age patients with severe sepsis treated in ICUs. Criteria for study entry included a diagnosis of severe sepsis defined as a suspected or proven infection and presence of one or more acute sepsis-induced organ dysfunctions, and treatment for severe sepsis in a participating ICU. Treatment was the standard of care at each participating ICU. Evaluations, procedures, or treatment beyond those used at each institution's standard of care were not performed. As a result, ethical review board approval and informed consent were not a uniform requirement, however, most countries obtained ethics review or approval to confirm that informed consent was not required. Data for routine clinical practice parameters were collected for qualifying patients. Clinical data collected, via a secure website, included patient demographics, co-morbid conditions, clinical features of severe sepsis patients, characteristics of infection, therapy and support care, and ICU outcomes. There were no study-specific interventions and no attempt was made to alter the treatment that patients received. The study was conducted at 276 study centers in 37 countries and data were collected from December 2002 until December 2005 with 12,570 adult patients with severe sepsis entered into the database. An independent international medical advisory board was involved in study development, decisions surrounding data use, and publications. The PROGRESS website was developed and maintained by Eli Lilly and Company. The Progress Advisory Board was responsible for the oversight of the publication of results from this study, and provided approval to access and retrieve data from the study database.

### Patients

Patients could be enrolled in the study only if they had a diagnosis of severe sepsis and were treated in the ICU. The definition of severe sepsis used in PROGRESS, previously described [21], included both proven or suspected infection based on clinical presentation, and presence of one or more acute organ dysfunctions. Organ dysfunctions definitions are listed in Additional file 1, Table S1. Although there was no age limit for participation in the PROGRESS study, this sub-study evaluates only adult patients ≥18 years of age. Patients were evaluated for use of low-dose corticosteroids (equivalent or lesser potency to hydrocortisone 50 mg/6 hourly plus 50 μg 9-alpha-fludrocortisone) for the treatment of severe sepsis and vasopressors (>5 μg/kg/minute of dopamine; any dose of epinephrine, norepinephrine, phenylephrine, vasopressin or milrinone) at any time in the ICU.

### Data collection

Data for each patient in the study were entered electronically by the participating physician or other investigative site personnel with an electronic data form via a dedicated, secure website. Patient identities were kept anonymous. Patients with records that remained incomplete due to data or technical limitations (n = 388) were not included in the reporting database. Safety information was not captured.

### Statistical methods and statistical analyses

The purpose of this sub-study is to describe the use of low-dose corticosteroids in adult patients with severe sepsis across ICUs globally, comparing baseline characteristics, as well as the hospital mortality rates in these patients. Patients who were identified as chronic steroids recipients, or who received high doses of steroids, were excluded from the sub-study.

Summary statistics for demographic and clinical characteristics, co-morbid conditions, and supportive care were compared for low-dose corticosteroids use versus non-low-dose corticosteroids use overall, and for patients with and without vasopressors. Continuous variables were compared across treatment groups using non-parametric analysis of variance (ANOVAs) and qualitative variables were compared using the chi-square test.

Because of the non-randomized nature of this observational study, there could be baseline imbalances between the low-dose corticosteroid and non-low-dose corticosteroid treatment groups. This could lead to bias estimates of the effect of low-dose corticosteroids on mortality unless methods are instituted to control for potential confounders. To implement these adjustments, a two-step bias-removing procedure was performed. The first step of this procedure was to estimate a propensity score for each subject using logistic regression of treatment received on covariates [23,24], with variables screened from the baseline characteristics. Covariates for potential inclusion in the propensity model were identified as candidate variables on the basis of univariate mortality analysis (see Additional file 2, Table S4). Any variable for which 20% or more of the patients had missing values was not included as candidates in the propensity score model. Twelve variables (age, seven types of ODs, surgical status, chronic lung disease status, active cancer status, and other chronic disabling condition) with *P*-values less than 0.10 were selected for the logistic propensity model. A patient's propensity score is the conditional probability of receiving low-dose corticosteroids given their observed values of the 12 selected predictors in the propensity score model. The propensity score is a single number which synthesizes the effect of the 12 covariants on the probability of receiving low-dose corticosteroids. Patients were subdivided into quintiles based on their propensity scores and the propensity score quintile was used in logistic regression models of mortality. Additional details and discussion concerning propensity score development can also be viewed in Additional file 2.

In the second step of the statistical adjustment process, a set of logistic models were developed to assess the effect of treatment (low-dose corticosteroid use; non-low-dose corticosteroid use) on hospital mortality. In addition to treatment, models included propensity score quintiles, and factors that were significantly associated with mortality as additional covariates. In these multivariate logistic models, adjusted odds ratios of the effect of low-dose corticosteroid treatment on hospital mortality with corresponding 95% confidence intervals, and *P*-values are presented.

To assess the degree to which the propensity score method was successful in the correction of the imbalance between the two treatment groups, the Kolmogorov-Smirnov test and the chi-square test were performed within each propensity score quintile, facilitating comparisons of the distributions of the continuous and qualitative variables within said quintiles. *P*-values for the propensity quintiles were tabulated alongside the *P*-values from the unadjusted baseline comparisons.

## Results

**Figure 1:**
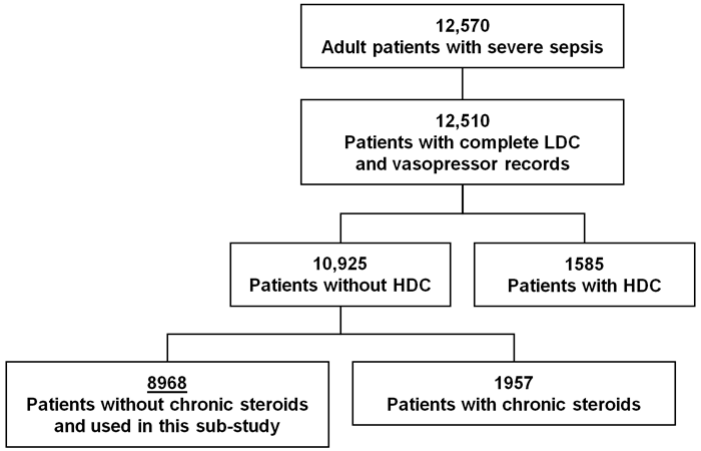
**Patient disposition**. Patients were enrolled from December 2002 until December 2005 in 37 countries at 276 sites. There were 12,570 adult patients with severe sepsis entered into the PROGRESS database of which 8,968 were used for this sub-study. LDC, Low-Dose Corticosteroids; HDC, High-Dose Corticosteroids.

A total of 12,570 adult patients with severe sepsis were entered into the PROGRESS study database. Of these patients, 12,510 had complete data for both low-dose corticosteroid and vasopressor use. Of these patients, 8,968 did not receive chronic or high-dose steroids, and made up the patient population described in this sub-study (Figure 1). Patients with high-dose corticosteroids use were excluded from the analysis as high-dose steroid administration confounds the specific assessment of low-dose corticosteroid use. Patients with chronic steroid use were also excluded to remove the possible confounder that these patients with potential chronic adrenal suppression may benefit from adrenal replacement therapy, independent of any specific effect on the treatment of septic shock (See Additional file 1, Table S2).

Regional and country-specific low-dose corticosteroid use data (from countries with patient enrollment >1% and >1% of total steroid use) are presented in Table 1. Regional use of low-dose corticosteroids was highest in Europe (51.1%; 1,116/2,184 patients) and lowest in Asia (21.6%; 549/2,547 patients). Country use was highest in Brazil (62.9%; 538/856 patients) and lowest in Malaysia (9.0%; 47/522 patients).

**Table 1:** Low-dose corticosteroid use by region and country

Region	Country*	Patients (N = 8,968) n (%)	Within Study LDC Use (N = 3,051) n (%)	Within Country LDC Use n (%)	Within Region LDC Use n (%)
**Europe†:**		2,184 (24.4)	1,116 (36.6)		1,116 (51.1)
	**Poland**	181 (2.0)	107 (3.5)	107 (59.1)	
	**Germany**	1,459 (16.3)	754 (24.7)	754 (51.7)	
	**Belgium**	238 (2.7)	91 (3.0)	91 (38.2)	
	**Other European Countries**	306 (3.4)	164 (5.4)	164 (53.6)	

**Latin America†:**		2,869 (32.0)	1,063 (34.8)		1,063 (37.1)
	**Brazil**	856 (9.5)	538 (17.6)	538 (62.9)	
	**Chile**	261 (2.9)	112 (3.7)	112 (42.9)	
	**Peru**	222 (2.5)	57 (1.9)	57 (25.7)	
	**Argentina**	973 (10.8)	249 (8.2)	249 (25.6)	
	**Mexico**	368 (4.1)	80 (2.6)	80 (21.7)	
	**Other Latin American Countries**	189 (2.1)	27 (0.9)	27 (14.3)	

**Northern America†:**		523 (5.8)	139 (4.6)		139 (26.6)
	**United States/(Canada**^1^**)**	523 (5.8)	139 (4.6)	139 (26.6)	

**Oceania†:**		683 (7.6)	156 (5.1)		156 (22.8)
	**Australia**	557 (6.2)	132 (4.3)	132 (23.7)	
	**Other Oceania Countries**	126 (1.4)	24 (0.8)	24 (19.0)	

**Asia†:**		2,547 (28.4)	549 (18.0)		549 (21.6)
	**India**	681 (7.6)	197 (6.5)	197 (28.9)	
	**Singapore**	188 (2.1)	41 (1.3)	41 (21.8)	
	**Philippines**	411 (4.6)	87 (2.9)	87 (21.2)	
	**Israel**	150 (1.7)	29 (1.0)	29 (19.3)	
	**Malaysia**	522 (5.8)	47 (1.5)	47 (9.0)	
	**Other Asian Countries**	595 (6.6)	148 (4.9)	148 (24.9)	

**Other Regions†:**		162 (1.8)	28 (0.9)	28 (17.3)	28 (17.3)

* With enrollment >1% patient population and >1% total steroid use.

† Countries from:

Europe: Germany, Belgium, Poland, Netherlands, Hungary, Romania, Austria and Slovak Republic.

Latin America: Argentina, Brazil, Mexico, Chile, Peru, Colombia, Venezuela and Puerto Rico.

Northern America: United States and Canada.

Oceania: Australia and New Zealand.

Asia: India, Malaysia, Philippines, Singapore, Israel, Taiwan, Thailand, Hong Kong, Turkey, Saudi Arabia, Lebanon, China and Kuwait.

Other Regions: Africa with Algeria, Egypt and South Africa as countries (patient population and total steroid use <1%).

^1 ^Only one patient with non-missing chronic steroid data from Canada, and included in the analyses.

LDC, low-dose corticosteroids.

Table 2 presents the baseline characteristics of PROGRESS adult patients with severe sepsis included in this sub-study, as well as vasopressor use. A total of 34.0% (3,051/8,968) of patients received low-dose corticosteroids and 79.8% (7,160/8,968) received vasopressors. In patients receiving vasopressors, 39.0% (2,794/7,160) received low-dose corticosteroids versus 14.2% (257/1,808) in patients who never received vasopressors. In all clinical characteristics shown, baseline imbalances were present between patients who received low-dose corticosteroids and those who did not, although the pattern of imbalances of baseline characteristics between LDC and non-LDC patients sometimes differed in patients not receiving vasopressors compared to those receiving vasopressors. Patients receiving low-dose corticosteroids were older (mean age 62.4 versus 59.5 years), were more likely to have undergone surgery (45.0% versus 39.4%), had more co-morbidities, and greater disease severity scores (SOFA - Sequential Organ Failure Assessment) score, 10.1 versus 8.6 and APACHE II (Acute Physiology and Chronic Health Evaluation II) score 24.7 versus 22.1) than patients who never received low-dose corticosteroids. The number of organ dysfunctions (OD) in the low-dose corticosteroids group was 3.9 versus 3.2 in the non-low-dose corticosteroids group.

**Table 2:** Patient baseline characteristics

	VASOPRESSOR-YES (N = 7,160)			VASOPRESSOR-NO (N = 1,808)			TOTAL (N = 8,968)		
			
	LDC (N = 2,794)	Non-LDC (N = 4,366)	*P*-value	LDC (N = 257)	Non-LDC (N = 1,551)	*P*-value	LDC (N = 3,051)	Non-LDC (N = 5,917)	*P*-value
**Age, mean (SD)**	62.8 (16.3)	60.5 (17.9)	<0.001	59.1 (19.0)	56.6 (19.2)	0.049	62.4 (16.6)	59.5 (18.3)	<0.001
**Region, n (%)**									
**Europe**	1,067 (38.2)	878 (20.1)	<0.001	49 (19.1)	190 (12.3)	<0.001	1,116 (36.6)	1,068 (18.0)	<0.001
**Latin America**	992 (35.5)	1,174 (26.9)	<0.001	71 (27.6)	632 (40.7)	<0.001	1,063 (34.8)	1,806 (30.5)	<0.001
**Northern America**	112 (4.0)	285 (6.5)	<0.001	27 (10.5)	99 (6.4)	<0.001	139 (4.6)	384 (6.5)	<0.001
**Oceania**	150 (5.4)	429 (9.8)	<0.001	6 (2.3)	98 (6.3)	<0.001	156 (5.1)	527 (8.9)	<0.001
**Asia**	450 (16.1)	1,486 (34.0)	<0.001	99 (38.5)	512 (33.0)	<0.001	549 (18.0)	1,998 (33.8)	<0.001
**Other Regions**	23 (0.8)	114 (2.6)	<0.001	5 (1.9)	20 (1.3)	<0.001	28 (0.9)	134 (2.3)	<0.001
**Surgical, n (%)**	1,309 (46.9)	1,795 (41.1)	<0.001	64 (24.9)	535 (34.5)	0.002	1,373 (45.0)	2,330 (39.4)	<0.001
**Cardiovascular, n (%)**	2,579 (92.3)	3,828 (87.7)	<0.001	49 (19.1)	333 (21.5)	0.346	2,628 (86.1)	4,161 (70.3)	<0.001
**Respiratory, n (%)**	2,445 (87.5)	3,587 (82.2)	<0.001	225 (87.5)	1,192 (76.9)	<0.001	2,670 (87.5)	4,779 (80.8)	<0.001
**Hematology, n (%)**	1,086 (38.9)	1,468 (33.6)	<0.001	94 (36.6)	389 (25.1)	<0.001	1,180 (38.7)	1,857 (31.4)	<0.001
**Renal, n (%)**	1,568 (56.1)	1,981 (45.4)	<0.001	68 (26.5)	487 (31.4)	0.098	1,636 (53.6)	2,468 (41.7)	<0.001
**Hepatic, n (%)**	675 (24.2)	893 (20.5)	0.001	35 (13.6)	232 (15.0)	0.512	710 (23.3)	1125 (19.0)	<0.001
**Metabolic, n (%)**	1,477 (52.9)	1,923 (44.0)	<0.001	95 (37.0)	452 (29.1)	0.023	1,572 (51.5)	2,375 (40.1)	<0.001
**CNS, n (%)**	1,138 (40.7)	1,417 (32.5)	<0.001	86 (33.5)	502 (32.4)	0.952	1,224 (40.1)	1,919 (32.4)	<0.001
Number of OD, mean (SD)*	4.0 (1.5)	3.5 (1.5)	<0.001	2.6 (1.4)	2.3 (1.3)	0.001	3.9 (1.6)	3.2 (1.5)	<0.001
**SOFA, mean (SD)**	10.4 (3.6)	9.7 (3.6)	<0.001	6.1 (2.8)	5.2 (3.0)	0.013	10.1 (3.7)	8.6 (4.0)	<0.001
**APACHE II, Mean (SD)**	25.2 (8.0)	23.1 (8.2)	<0.001	18.8 (6.7)	19.1 (7.0)	0.688	24.7 (8.1)	22.1 (8.1)	<0.001
**Chronic Lung Disease, n (%)**	486 (17.4)	541 (12.4)	<0.001	73 (28.4)	199 (12.8)	<0.001	559 (18.3)	740 (12.5)	<0.001
**Active Cancer, n (%)**	499 (17.9)	649 (14.9)	<0.001	33 (12.8)	174 (11.2)	0.455	532 (17.4)	823 (13.9)	<0.001
**Other Chronic Disabling Condition, n (%)**	725 (25.9)	857 (19.6)	<0.001	56 (21.8)	316 (20.4)	0.660	781 (25.6)	1173 (19.8)	<0.001
**Fungal Infection, n (%)**	352 (12.6)	346 (7.9)	<0.001	14 (5.4)	87 (5.6)	0.735	366 (12.0)	433 (7.3)	<0.001

* Patients with missing data on one or more organs were excluded from the organ dysfunction summary.

P-values are from a chi-square test for categorical variables, and from a non-parametric ANOVA test for continuous variables.

LDC = Low-dose Corticosteroids, SD = Standard Deviation, CNS = Central Nervous System, OD = Organ Dysfunction, SOFA = Sequential Organ Failure Assessment, APACHE = Acute Physiology and Chronic Health Evaluation.

A description of the intensive care therapies that patients received is given in Table 3. Significant differences exist between therapies received in all patients receiving low-dose corticosteroids versus those not receiving low-dose corticosteroids, except for mechanical venous thromboembolism (VTE) prophylaxis. Patients receiving low-dose corticosteroids received more therapeutic organ support and specific severe sepsis therapies, including drotrecogin alfa (activated) (DAA). In general, these differences were most marked in those receiving vasopressors. Intravenous (IV) fluid resuscitation was given to 94.7% (2,645/2,794) of low-dose corticosteroids patients on vasopressors and 67.7% (174/257) of low-dose corticosteroids patients not receiving vasopressors. Patients receiving low-dose corticosteroids spent longer in ICU than patients not on low-dose corticosteroids (median of 12 versus 8 days; *P *<0.001), and spent more days on vasopressors (median of 6 versus 3; *P *<0.001), as shown in Table 4.

**Table 3:** Patient therapies

	VASOPRESSOR-YES (N = 7,160)			VASOPRESSOR-NO (N = 1,808)			TOTAL (N = 8,968)		
			
Variable, n (%)	LDC (N = 2,794)	Non-LDC (N = 4,366)	*P*-value	LDC (N = 257)	Non-LDC (N = 1,551)	*P*-value	LDC (N = 3,051)	Non-LDC (N = 5,917)	*P-*value
**IV Fluid Resuscitation**	2,645 (94.7)	3,944 (90.3)	<0.001	174 (67.7)	1,022 (65.9)	0.579	2,819 (92.4)	4,966 (83.9)	<0.001
**Mechanical Ventilation**	2,628 (94.1)	3,809 (87.2)	<0.001	173 (67.3)	934 (60.2)	0.031	2,801 (91.8)	4,743 (80.2)	<0.001
**Nutrition: Enteral**	2,133 (76.3)	2,984 (68.3)	<0.001	181 (70.4)	1,125 (72.6)	0.473	2,314 (75.8)	4,109 (69.4)	<0.001
**Nutrition: Parenteral**	1,293 (46.3)	1,350 (30.9)	<0.001	106 (41.2)	295 (19.0)	<0.001	1,399 (45.9)	1,645 (27.8)	<0.001
**Heparin: LMW**	1,203 (43.1)	1,552 (35.6)	<0.001	119 (46.3)	455 (29.3)	<0.001	1,322 (43.3)	2,007 (33.0)	<0.001
**Heparin: Unfractionated**	1,287 (46.1)	1,452 (33.3)	<0.001	48 (18.7)	454 (29.3)	<0.001	1,335 (43.8)	1,906 (32.2)	<0.001
**Mechanical VTE Prophylaxis**	671 (24.0)	1116 (25.6)	0.091	77 (30.0)	234 (15.1)	<0.001	748 (24.5)	1,350 (22.8)	0.107
**Renal Replacement Therapy**	875 (31.3)	839 (19.2)	<0.001	20 (7.8)	142 (9.2)	0.474	895 (29.3)	981 (16.6)	<0.001
**Platelet Transfusion**	579 (20.7)	696 (15.9)	<0.001	22 (8.6)	82 (5.3)	0.037	601 (19.7)	778 (13.2)	<0.001
**DAA Therapy**	313 (11.2)	234 (5.4)	<0.001	13 (5.1)	23 (1.7)	<0.001	326 (10.7)	257 (4.3)	<0.001

*P*-values are from the Chi-Square test.

LDC, low-dose corticosteroids, IV, intravenous, LMW, low-molecular weight, VTE, venous thromboembolism, DAA, Drotrecogin alfa (activated).

**Table 4:** Number of days spent in ICU and days on low-dose corticosteroids and vasopressers during ICU stay

		VASOPRESSOR-YES (N = 7,160)			VASOPRESSOR-NO (N = 1,808)			TOTAL (N = 8,968)		
				
	Statistic	LDC (N = 2,794)	Non-LDC (N = 4,366)	*P*-value	LDC (N = 257)	Non-LDC (N = 1,551)	*P*-value	LDC (N = 3,051)	Non-LDC (N = 5,917)	*P*-value
**Days in ICU**	n	2,771	4,285	<0.001	255	1,532	0.017	3,026	5,817	<0.001
	Median	13	9		8	7		12	8	
	10^th ^percentile	3	2		3	2		3	2	
	25^th ^percentile	6	4		5	4		6	4	
	75^th ^percentile	24	18		15	12		23	16	
	90^th ^percentile	39	29		28	21		38	27	

**Days on LDC**	n	2,371	916	<0.001	244	257	<0.001	2,615	1,173	<0.001
	Median	6	----		5	----		6	----	
	10^th ^percentile	2	----		2	----		2	----	
	25^th ^percentile	3	----		3	----		3	----	
	75^th ^percentile	10	----		9	----		10	----	
	90^th ^percentile	16	----		15	----		16	----	

**Days on Vasopressors**	n	2,390	3,819	0.001	43	261	1.00	2,433	4,080	<0.001
	Median	6	4		----	----		6	3	
	10^th ^percentile	2	1		----	----		2	1	
	25^th ^percentile	3	2		----	----		3	2	
	75^th ^percentile	11	7		----	----		11	7	
	90^th ^percentile	18	12		----	----		18	12	

P-values are from a non-parametric ANOVA test.

LDC = Low-dose Corticosteroids, ICU = Intensive Care Unit.

Table 5 presents a summary of the mortality data. Hospital mortality with and without low-dose corticosteroids treatment was 60.8% (1,608/2,646) and 49.8% (2,042/4,101; *P *<0.001), respectively, in patients receiving vasopressors and 27.4% (66/241) and 23.9% (353/1,475; *P *= 0.248), respectively, in patients not receiving vasopressors. All patient mortality rates were greater in the low-dose corticosteroids group than in the non-low-dose corticosteroids group at 58.0% (1,674/2,887) versus 43.0% (2,395/5,576; *P *<0.001).

**Table 5:** Hospital mortality

Vasopressor	Hospital Mortality	LDC Use	Non-LDC Use	Odds Ratio (95% CI)	*P*-value
**Yes**	**No. of Patients Died (%)**	1,608/2646 (60.8%)	2,042/4,101 (49.8%)	1.56 (1.41, 1.72)	<0.0001
**No**	**No. of Patients Died (%)**	66/241 (27.4%)	353/1,475 (23.9%)	1.20 (0.88, 1.63)	0.2477
All Patients*	**No. of Patients Died (%)**	1,674/2887 (58.0%)	2,395/5,576 (43.0%)	1.83 (1.67, 2.01)	<0.0001

Analysis Population N = 8,968.

* 505 patients with missing hospital status were excluded from the analysis. Hospital mortality is defined as died in hospital or ICU.

*P*-value is from a logistic model: Mortality, LDC (yes/no). The Odds Ratio compares LDC versus Non-LDC patients.

LDC, Low-Dose Corticosteroids, CI, Confidence Intervals, ICU, Intensive Care Unit.

Because of the noted imbalances in baseline characteristics (greater age, regional use, co-morbidities, disease severity, and requirement for organ support) between those with and without low-dose corticosteroids therapy, mortality results for the two cohorts are not directly comparable. Therefore, multiple logistic regression models were developed utilizing propensity scores and independent mortality risk factors in an attempt to ameliorate the impact of observed differences between the two cohorts in this non-randomized comparison. Eleven models are presented in Table 6. These models began with one covariate, propensity quintiles (Model 1) based on 12 baseline characteristics (age, seven types of ODs (seven ODs), surgical status, chronic lung disease status, active cancer status, and other chronic disabling condition). Model 3, considered the core model, includes the propensity score quintiles as well as key covariates used to calculate the propensity quintiles; Age; and seven ODs. Model 3 was considered the core model as it contains prognostic characteristics highly predictive of outcomes in sepsis. A model without propensity quintiles (Model 2) was also included to assess the effect of core model components, age and seven ODs, on mortality. In Models 4-7, the effect of the core model on selected subsets of patients is evaluated. In Models 8-11, additional factors (Source of Infection, Number of Organ Dysfunctions, Active Cancer, APACHE II scores, Surgical Status, Vasopressors and Country) associated with mortality based on their association by univariate analysis (Additional file 2, Table S4) are added to the core model (with further evaluation of patient subgroups in Models 9 and 10).

**Table 6:** Summary of multivariate logistic regression models for hospital mortality

Model Number	Hospital Mortality Adjusted for	N Used/Read in Model*	R-Square	Goodness of Fit **Chi-Square**^†^	LDC Effect Chi-Square	Odds Ratio Point Estimate (95% CI)
**1**	Propensity Quintiles^1^	6,833/ 8,968	0.131	0.874	<0.0001	1.398 (1.256, 1.556)
**2**	Age, 7 OD	7,289/ 8,968	0.187	0.202	<0.0001	1.382 (1.244, 1.536)
**3**	Age, Propensity Quintiles, 7 OD (CORE MODEL)	6,833/8,968	0.186	0.296	<0.0001	1.392 (1.247, 1.553)
**4**	CORE MODEL, including Vasopressor-yes data only	5,403/7,160	0.158	0.814	<0.0001	1.328 (1.180, 1.494)
**5**	CORE MODEL, including Vasopressor-no data only	1,430/1,808	0.124	0.260	0.5456	1.115 (0.784, 1.585)
**6**	CORE MODEL, excluding DAA use	6,330/8,345	0.194	0.511	<0.0001	1.470 (1.310, 1.650)
**7**	CORE MODEL, including HDC use	7,519/10,925	0.184	0.619	<0.0001	1.369 (1.235, 1.517)
**8**	CORE MODEL, Source of Infection, Active Cancer, APACHE II Scores^2^, Surgical Status, Vasopressors	4,995/8,968	0.264	0.154	0.0001	1.301 (1.138, 1.487)
**9**	Age, Propensity Quintiles, Source of Infection, Active Cancer, APACHE II Scores^2^, Number of organ dysfunctions, including vasopressor-yes only data	3,955/7,160	0.201	0.5503	<0.0001	1.349 (1.173, 1.551)
**10**	Age, Propensity Quintiles, Source of Infection, Active Cancer, APACHE II Scores^2^, Number of Organ Dysfunctions, including vasopressor-no data only	1,040/1,808	0.218	0.670	0.4335	1.194 (0.766, 1.860)
**11**	Logistic regression with the 12 variables used in the Propensity Score Model and Country^3^	6,833/8,968	0.197	0.082	<0.0001	1.414 (1.252, 1.598)

* Includes only patients with severe sepsis and complete vasopressor and low-dose corticosteroid use. Excludes patients with high-dose and chronic corticosteroid use.

^† ^*P*-value from the Hosmer and Lemeshow Goodness-of-Fit Test.

^1 ^Propensity Quintiles based on age, 7 OD (cardiovascular, respiratory, renal, hematology, hepatic, metabolic, neurological), Surgical Status, Chronic Lung Disease Status, Active Cancer Status, and other Chronic Disabling Condition.

^2 ^APACHE II Scores in models were not imputed to the mean.

^3 ^Countries included: Belgium, Germany, Poland, Europe Others, Argentina, Brazil, Chile, Colombia, Latin America Others, Mexico, Peru, Australia, Oceania Others, Asia Others, India, Israel, Malaysia, Philippines, Taiwan, and Thailand.

LDC, Low-Dose Corticosteroids; CI, Confidence Intervals; OD, Organ Dysfunction; DAA, Drotrecogin Alfa (Activated); HDC, High-Dose Corticosteroids; APACHE, Acute Physiology and Chronic Health Evaluation.

All models applied to the study population (with vasopressor use) showed a consistent and significant association between low-dose corticosteroids and hospital mortality with odds ratios varying from 1.301 (1.138 to 1.487, 95% CI) in Model 8 to 1.470 (1.310 to 1.650, 95% CI), in Model 6. The exceptions are Models 5 and 10, based only on the sub-populations of patients who did not receive vasopressors, with an odds ratio of 1.115 (0.784 to 1.585, 95% CI) and 1.194 (0.766 to 1.860, 95% CI), respectively. This result is consistent with the unadjusted mortality results from Table 5 where the difference between the low-dose corticosteroids use and non-low-dose corticosteroids use in the non-vasopressors group was small (27.4% versus 23.9%) and not statistically significant (*P *= 0.248). It is interesting to note that the odds ratios were very similar in models with fewer factors (for example, Model 3) and in models with more factors included (for example, Models 9 and 11). All models also showed non-significant *P*-values (*P *> 0.05) for the Hosmer and Lemeshow Goodness of Fit test, indicating that there is insufficient evidence to reject the logistic regression models for lack-of-fit even in a very large dataset, thus implying that the models provide adequate fits to the data. Within each propensity score quintile, mortality was always higher in the low-dose corticosteroid group than in the non-low-dose corticosteroid group, with an increasing mortality trend across the propensity score quintiles (see Additional file 1, Table S3).

**Figure 2:**
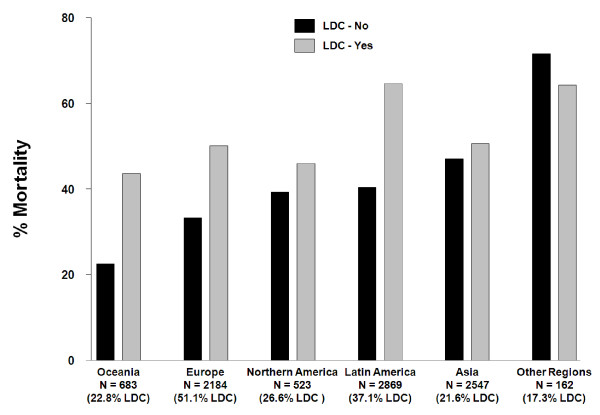
**Comparison of within-region mortality**. N, the number of patients within the region, and the % of LDC represents the percentage of low-dose corticosteroid use within the region.

Given the large regional and country variation in low-dose corticosteroids use and relative mortality rates, regional mortality comparisons are shown with low-dose corticosteroid and non-low-dose corticosteroid use by region. Results are indicated in Figure 2 and demonstrate that a similar trend between regions exists with percentage mortality levels higher in the low-dose corticosteroid use group, apart from the *Other Region *group containing a small sample size (n = 162) and the least low-dose corticosteroid use (17.3%).

**Figure 3:**
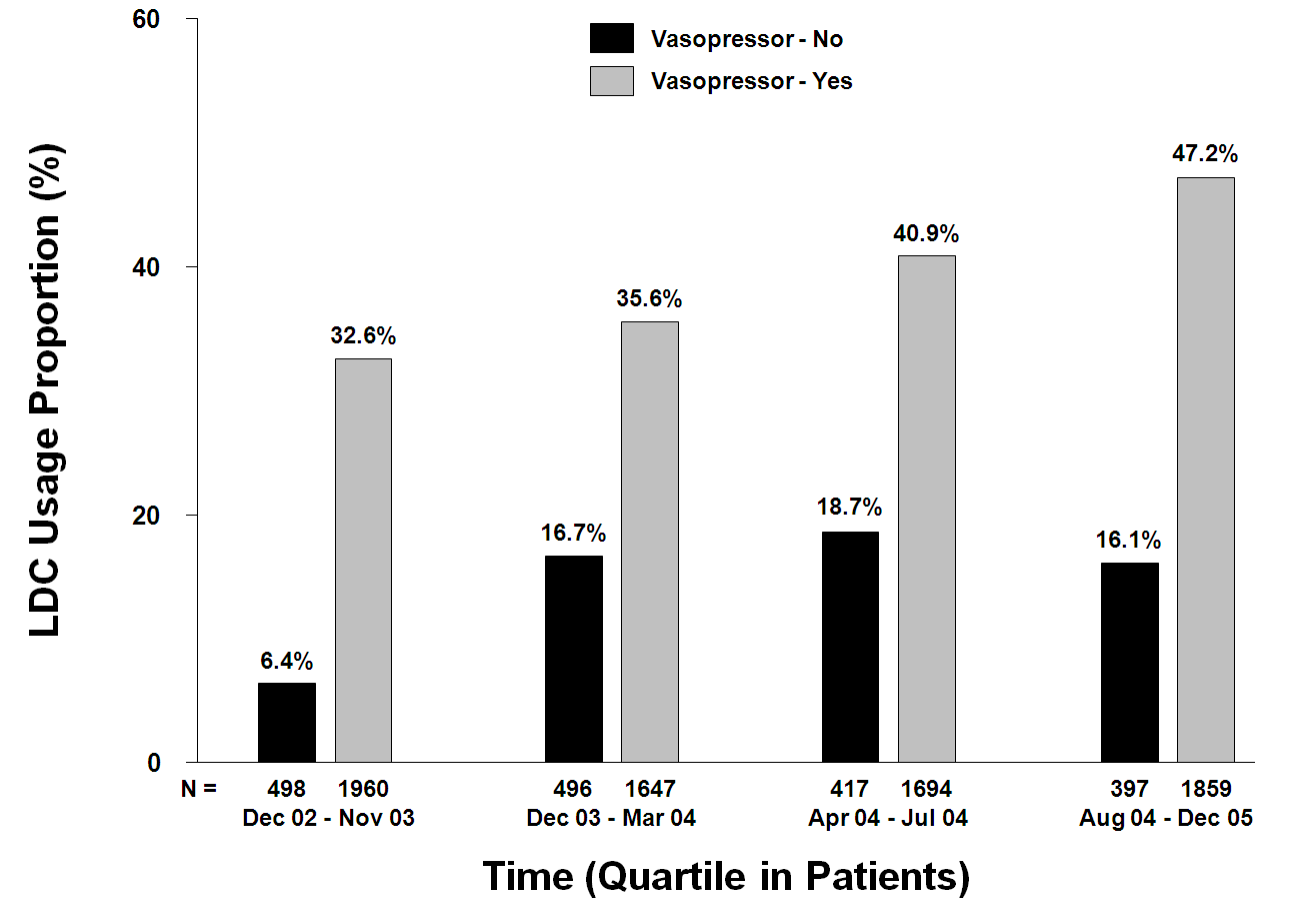
**Temporal pattern of low-dose corticosteroid use over time**. N, the number of patients in the quartiles.

Figure 3 shows the temporal pattern of low-dose corticosteroids usage from December 2002 to December 2005 in patient quartiles. The rate of low-dose corticosteroids usage in conjunction with vasopressors, has steadily increased over time from approximately 33% to 47%. In patients without vasopressors, the low-dose corticosteroids usage rate increased from 6.4% to 16.7% between the December 2002 to November 2003 and December 2003 to March 2004 timeframe, and remained relatively steady thereafter (between 16.1% and 18.7%).

## Discussion

PROGRESS is one of the largest global severe sepsis registries ever completed with 12,570 adult patients in 37 countries identified as having severe sepsis. Given the recent controversy over the use of low-dose corticosteroids for this deadly disease, our study provides important novel information on the use of low-dose corticosteroids in everyday clinical practice over several years, in addition to providing information on treatment variation across regions and countries. These results indicate widespread adoption of low-dose corticosteroids for the treatment of severe sepsis with significant regional and country variation, and increased hospital mortality in patients treated with low-dose corticosteroids, even after adjustment for baseline imbalances in disease severity.

The emerging picture from the PROGRESS registry regarding low-dose corticosteroids use is an important one particularly when the controversy on their use remains present after contradictory results from trials, guidelines and meta-analyses [25-28]. Hitherto there has been a paucity of data regarding global practice of the usage of low dose corticosteroids in severe sepsis. There appears to be regional variation in their use with the highest rate reported in Europe. Even within regions, a large difference is observed, for example, within Latin America, low-dose corticosteroids use ranges from 62.9% (in Brazil) to 21.7% (in Mexico).

It is likely that the use of low-dose corticosteroids increased in clinical practice following the publication of the Annane *et al*. 2002 steroids results [8] and their subsequent recommended use in the 2004 SSC guidelines [9], although published data are lacking. A study in Slovakia [29] did find that low-dose corticosteroid use increased by 49.2% in 2006 compared to 2004. Our results do confirm that global low-dose corticosteroids use has increased steadily over time from 2002 to 2005 in patients receiving vasopressors. In patients not receiving vasopressor treatment, low-dose corticosteroid use was seen to drop slightly in the last year of enrollment. When considering how evolving practice and the introduction of the first set of Surviving Sepsis Campaign guidelines in March 2004 [9] may have influenced the use of low-dose corticosteroids, it is important to note that recruitment in PROGRESS in many countries was largely completed by the end of 2004, and therefore will have limited ability to detect the effect of these guidelines. It may be interesting to evaluate the use of corticosteroids for severe sepsis and septic shock patients from 2004 until now given the CORTICUS study [14] and new guidelines [15].

The present sub-study also demonstrates significant differences in most baseline characteristics between patients receiving low-dose corticosteroids and those that did not. Low-dose corticosteroid-treated patients were older, more likely to have undergone surgery (if receiving vasopressor therapy), have more organ dysfunction, and receive higher levels of therapeutic organ support and severe sepsis therapies, including drotrecogin alfa (activated). Patients receiving low-dose corticosteroids also received vasopressors for longer and spent longer in ICU than patients who did not receive low-dose corticosteroids. These differences point to a greater severity of illness in patients treated with low-dose corticosteroids. This was also seen from the results of steroid use in the PROWESS trial [30] which indicated that patients at high risk of death were more likely to be treated with corticosteroids.

In-hospital mortality was higher in the low-dose corticosteroid group. Other epidemiological studies [30,14] have noted higher use of steroids in sicker patients and may account for some of the higher mortality observed. When adjusted for imbalances by logistic modeling, low-dose corticosteroids patients still had significantly higher odds of death. In terms of model development, given that data collection was not complete for all parameters, the intent was to develop a simple model that provided relevant clinical data for a high percentage of patients and to assess the model performance as more clinical characteristics were added. Many baseline imbalances were clinically relevant, and population quintiles, based on a propensity model, were developed to address these imbalances. It should be noted that the model used baseline characteristics for adjustment, however, corticosteroid use could be at any time in the ICU stay, and therefore, it is possible that the baseline characteristics do not reflect the characteristics of the patient treated when they received therapy. APACHE II Scores, due to large numbers of missing data, and regional differences were not in the propensity model or core mortality model. However, when they were added for comparative purposes in further mortality models, the conclusion of the effect of low-dose corticosteroids on mortality did not change. A number of additional mortality models were developed and presented, emphasizing the consistency of the estimates of the effect of low-dose corticosteroids across models of different sizes and with different combinations of covariates. The conclusion from this modeling was that low-dose corticosteroid treatment was associated with increased mortality regardless of the model used, when adjusted by relevant clinical and demographic factors. This observation of higher mortality with low-dose corticosteroids after adjustment for disease severity is consistent with a previous study [13]. A recent prospective, multi-center, observational study of 2,796 patients to analyze the effectiveness of treatments recommended in the sepsis guidelines using propensity scores [19], found no benefits in administration of low-dose corticosteroids in severe sepsis. These results of the Ferrer *et al*. 2009 study [19] agree with the findings of CORTICUS [14]. Analysis of the pivotal trials in severe sepsis using Bayesian methodology reached very similar results, showing no benefit with low-dose corticosteroids [20]. In contrast, two recent meta-analyses of randomized clinical trial results, [16,17] demonstrated significant reduction in 28-day all cause mortality (*P *= 0.02) and hospital mortality (*P *= 0.05) with low-dose corticosteroids given for ≥5 days [16], and in a subgroup of trials published after 1997, steroids were found to be harmful in less severely ill patient populations and beneficial in more severely ill patient populations [17], with the effects of low-dose corticosteroids on mortality appearing to be dependent on severity of illness.

An important finding of this study was the relatively high incidence of low-dose corticosteroid use (14.2%) in patients with severe sepsis which did not require vasopressor agents. It is likely that the low cost of corticosteroids and physician comfort prescribing this therapy are significant factors in this inappropriate usage of low dose corticosteroids. Recommendations have stated that these patients should not receive steroids [8,15]. Because of the potential complications of corticosteroids, especially superinfection [1,13], physicians should use steroids only in those patients who have clear indications for their use. In CORTICUS [14], a trend towards increased superinfection was noted among patients who received hydrocortisone (OR = 1.27; 95% CI: 0.96 to 1.68). Interestingly, the recent Annane *et al*. 2009 meta-analysis [16] showed no evidence of increased risk of gastroduodenal bleeding, superinfection, or acquired neuromuscular weakness with low-dose corticosteroids; however, their use was associated with an increased risk of developing hyperglycemia and hypernatremia. Unfortunately, this large registry did not collect safety information, thus we cannot examine possible side effects of corticosteroids in our population.

The strengths of this study include the large number of prospectively enrolled patients in many countries and reflected *real world *clinical practice. It also included rigorous statistical model development, including propensity scores, to try and compensate for observed differences in disease severity. As with any observational study, there are inherent weaknesses with the PROGRESS study. The lack of a randomized control group clearly sets limitations to any inferences that might be drawn about the effect of low-dose corticosteroid use on clinical outcomes and the study was not prospectively designed to examine mortality by low-dose corticosteroid use. Other limitations include the fact that the PROGRESS registry was designed to describe global patterns of care in severe sepsis, not an assessment of low-dose corticosteroid use, and the exact timing, dose and duration of low-dose corticosteroid treatment and adrenal status was not assessed. Similarly, the exact timing, dose and duration of vasopressor treatment was also not recorded. It was perhaps surprising that fluid resuscitation was not reported in 32% of the vasopressor-no group receiving low-dose corticosteroids, raising the possibility that although sites were requested to record 'low dose steroids as treatment for severe sepsis' that in fact low-dose corticosteroid treatment was not being used as a specific treatment of severe sepsis in some patients. However, in the models including only vasopressor-no patients (Models 5 and 10), results were consistent with other models suggesting that the overall results were not driven by the vaspressor-no group. Although PROGRESS involved large patient numbers, significant levels of missing values resulted in small numbers for certain parameters. Country variability in the standard of care, the severity of cardiovascular organ dysfunction (except vasopressor requirements), and some characteristics, such as timing of organ dysfunction and of various treatments received, were not evaluated. Also relevant is that the various centers and countries were involved in the three-year study over different periods of time, which could have affected the standard of care with evolving practice (for example, SSC guidelines [9]). Regarding site selection, sites within a country were not randomly selected and so it is possible that site practice may not fully reflect the practice within that country, particularly for countries with relatively few sites. It is also difficult to explicitly determine to what extent the observed geographic variations in mortality resulted from the differences in baseline characteristics of the patients entered, differences in received therapies, genetic components, or other unrecorded factors. Finally, due to the observational nature of the study, adverse events and safety events were not recorded.

## Conclusions

The PROGRESS registry has helped document information on the use of low-dose corticosteroids in severe sepsis in everyday clinical practice and on treatment variation across regions and countries. Approximately 14% of severe sepsis patients received low-dose corticosteroids despite never receiving vasopressors during their ICU stay. Low-dose corticosteroids patients were older, had more co-morbidities and higher disease severity scores. When adjustments were made for imbalances, mortality remained significantly higher in the group of patients receiving low-dose corticosteroids.

## Key messages

• There is widespread adoption of low-dose corticosteroids for the treatment of severe sepsis with significant regional and country variation.

• Approximately 14% of severe sepsis patients received low-dose corticosteroids despite never receiving vasopressors during their ICU stay.

• PROGRESS registry patients treated with low-dose corticosteroids were older, had more co-morbidities and higher disease severity scores.

• Mortality was higher, and remained higher after adjusting for key determinants of mortality, in the low-dose corticosteroids group.

• Low-dose corticosteroids should not be used without careful patient selection.

## Abbreviations

ANOVA: analysis of variance; APACHE II: Acute Physiology and Chronic Health Evaluation II; DAA: Drotrecogin alfa (activated); ICU: intensive care unit; LDC: low-dose corticosteroids; MAP: mean arterial pressure; OD: organ dysfunction; PROGRESS: Promoting Global Research Excellence in Severe Sepsis; SBP: systolic blood pressure; SOFA: Sequential Organ Failure Assessment; SSC: Surviving Sepsis Campaign; VTE: venous thromboembolism.

## Competing interests

Drs. Beale, Levy, Martin and Profs. Dobb, Ramsay, Vallet, Vincent and Reinhart have all served as consultants to and participated in Eli Lilly and Company sponsored trials. Prof. Brunkhorst received research grants from Eli Lilly Deutschland GmbH. Dr. Martin's institution received funding for Dr. Martin to conduct clinical trials with Eli Lilly and Company. Dr. Silva has served as a consultant for Eli Lilly, Brazil, and Prof. Sprung has received lecture fees and served as a consultant for Eli Lilly and Company. Drs. Costigan, Leishman, Williams and Janes are employees and stockholders of Eli Lilly and Company.

## Authors' contributions

RB, FB, GD, ML, GM, GR, ES, BV, J-LV, MW and KR participated in the conception and design of the PROGRESS registry. RB, JJ, CS, TC and KR contributed to the development and conduct of the principle analyses in this sub-study. All authors contributed to drafting and critically revising the manuscript and read and approved the final version of the manuscript.

## Supplementary Material

Additional file 1 **Supplementary tables S1 to S3**. A Word file containing further clinical information, and the following tables: Table S1: Organ Dysfunction Definitions; Table S2: Chronic Steroids, High-Dose Corticosteroid and Drotrecogin Alfa (Activated) Patient Hospital Mortality Outcomes for Low-Dose Corticosteroid use (yes/no) and Vasopressor use (yes/no); Table S3: In Hospital Mortality Rates Across the Propensity Quintiles. Click here for file

Additional file 2 **Supplementary tables S4 to S5**. A Word file containing further statistical information on Propensity Score and Model Development and the following tables: Table S4: Summary of Baseline Characteristics Used in Mortality Models and Associated Hospital Mortality, a Univariate Analysis; Table S5: Improvement in Baseline Imbalance Using Propensity Score Analysis. Click here for file
