# Supplementary material for: Global utilization of low-dose corticosteroids in severe sepsis and septic shock: a report from the PROGRESS registry
Source: Crit Care. 2010 Jun 3;14(3):R102. doi: 10.1186/cc9044 (PMC2911744; doi:10.1186/cc9044)
Supplement: Additional file 1 — Supplementary tables S1 to S3. A Word file containing further clinical information, and the following tables: Table S1: Organ Dysfunction Definitions; Table S2: Chronic Steroids, High-Dose Corticosteroid and Drotrecogin Alfa (Activated) Patient Hospital Mortality Outcomes for Low-Dose Corticosteroid use (yes/no) and Vasopressor use (yes/no); Table S3: In Hospital Mortality Rates Across the Propensity Quintiles. [file cc9044-S1.DOCX]

**ADDITIONAL DATA FILE 1**

**Clinical Appendix**

**ADDITIONAL DATA FILE 1, TABLE S1: Organ Dysfunction Definitions**

| **Cardiovascular:** Hypotension in the absence of causes other than sepsis. For example, an arterial systolic blood pressure (SBP) of ≤90 mm Hg, a mean arterial pressure (MAP) ≤70 mm Hg for at least 1 hour despite adequate fluid resuscitation, >40 mm Hg drop in SBP from baseline, or the need for vasoactive agents to maintain SBP ≥90 mm Hg or MAP ≥70 mm Hg.  **Respiratory:** Acute lung injury due to sepsis and associated with serious hypoxemia. For example, O2 saturation <90% on room air, PaO2 ≤70 mm Hg, or PaO2/FiO2 ≤280.  **Renal:** Oliguria (average urine output <0.5 mL/kg/h for 1 hour despite adequate fluid resuscitation, <30 mL/h for 3 hours, or <700 mL/24 hours) or the need for renal replacement therapy as a result of severe sepsis.  **Hematology:** Thrombocytopenia, for example, platelet count <100,000/mm3 or a 50% decrease in platelet count from the highest value recorded over the last 3 days.  **Unexplained metabolic acidosis:** Defined by (1) pH ≤7.30 or base deficit ≥5.0 mEq/L and (2) a plasma lactate level >1.5 times the upper limit of normal for the reporting laboratory. Measurement of pH or base deficit and lactate level should occur within a clinically relevant time interval such that a causal relationship exists between the measured values.  **Neurological:** Evidence of encephalopathy with, for example, a Glasgow Coma score <13.  **Hepatic:** Markedly increased serum bilirubin level or jaundice. |
| --- |

**ADDITIONAL DATA FILE 1, TABLE S2: Chronic Steroids, High-Dose Corticosteroids, and Drotrecogin Alfa (Activated) Patient Hospital Mortality Outcomes for Low-Dose Corticosteroids use (yes/no) and Vasopressor use (yes/no) Patients**

|  | VASOPRESSOR-YES | | VASOPRESSOR-NO | |
| --- | --- | --- | --- | --- |
|  | LDC | Non-LDC | LDC | Non-LDC |
| DAA-Yes, n (%) | 231/480 (48.1%) | 160/344 (46.5%) | 6/19 (31.6%) | 2/35 (5.7%) |
| DAA-No, n (%) | 2027/3503 (57.9%) | 2695/5454 (49.4%) | 127/490 (25.9%) | 480/2110 (22.7%) |
| Chronic Steroids-Yes, n (%) | 307/511 (60.1%) | 239/372 (64.2%) | 27/104 (26.0%) | 42/127 (33.1%) |
| Chronic Steroids-No, n (%) | 1765/3106 (56.8%) | 2395/4954 (48.3%) | 80/305 (26.2%) | 382/1666 (22.9%) |
| High-Dose Steroids-Yes, n (%) | 238/473 (50.3%) | 500/806 (62.0%) | 32/103 (31.1%) | 54/197 (27.4%) |
| High-Dose Steroids-No, n (%) | 2031/3538 (57.4%) | 2360/5012 (47.1%) | 103/413 (24.9%) | 430/1962 (21.9%) |

LDC= Low-Dose Corticosteroids, DAA= Drotrecogin Alfa (Activated)

Table S2 is a summary table for chronic steroid, high-dose corticosteroids and drotrecogin alfa (activated) (DAA) patient’s hospital mortality outcomes for both low-dose corticosteroids and vasopressor use. This datum provides the rationale for excluding chronic steroid and high-dose corticosteroids patients from this sub-study. As the association between low-dose corticosteroids (yes/no) and mortality was directionally similar for both DAA yes and no patients, DAA patients were included in the sub-study. They were excluded in Model 6 as a sensitivity analysis.

**ADDITIONAL DATA FILE 1, TABLE S3: In Hospital Mortality Rates across the Propensity Quintiles**

|  | **LDC** | | **Non-LDC** | |  |  |
| --- | --- | --- | --- | --- | --- | --- |
| **Propensity Quintile** | **No. of Patients Died** | **% Mortality** | **No. of Patients Died** | **% Mortality** | **Odds Ratio**  **(95% CI)** | **P-value** |
| **1** | 75/241 | 31.1 | 286/1135 | 25.2 | 1.3  (1.0, 1.8) | 0.0577 |
| **2** | 154/380 | 40.5 | 302/958 | 31.5 | 1.5  (1.2, 1.9) | 0.0017 |
| **3** | 235/453 | 51.9 | 384/896 | 42.9 | 1.4  (1.1, 1.8) | 0.0017 |
| **4** | 348/567 | 61.4 | 454/808 | 56.2 | 1.2  (1.0, 1.5) | 0.0548 |
| **5** | 512/704 | 72.7 | 440/691 | 63.7 | 1.5  (1.2, 1.9) | 0.0003 |
