# Supplementary material for: Global utilization of low-dose corticosteroids in severe sepsis and septic shock: a report from the PROGRESS registry
Source: Crit Care. 2010 Jun 3;14(3):R102. doi: 10.1186/cc9044 (PMC2911744; doi:10.1186/cc9044)
Supplement: Additional file 2 — Supplementary tables S4 to S5. A Word file containing further statistical information on Propensity Score and Model Development and the following tables: Table S4: Summary of Baseline Characteristics Used in Mortality Models and Associated Hospital Mortality, a Univariate Analysis; Table S5: Improvement in Baseline Imbalance Using Propensity Score Analysis. [file cc9044-S2.DOCX]

**ADDITIONAL DATA FILE 2**

**Statistical Appendix**

**ADDITIONAL DATA FILE 2, TABLE S4: Summary of Baseline Characteristics Used in Mortality Models and Associated Hospital Mortality – a Univariate Analysis**

| **Variable** | **Died**  **(N=4069)** | **Alive**  **(N=4394)** | **Wald Chi-Square** | **P-value** |
| --- | --- | --- | --- | --- |
| **Total number of OD, Mean (SD)** | 4.0 (1.6) | 3.0 (1.4) | 702.469 | <0.001 |
| **APACHE II, Mean (SD)** | 25.9 (8.1) | 20.4 (7.4) | 618.990 | <0.001 |
| **Renal, n (%)** | 2301 (56.5) | 1585 (36.1) | 362.137 | <0.001 |
| **Metabolic, n (%)** | 2203 (54.1) | 1547 (35.2) | 308.882 | <0.001 |
| **Age, Mean (SD)** | 63.7 (16.7) | 57.3 (18.3) | 267.230 | <0.001 |
| **SOFA, Mean (SD)** | 10.2 (4.0) | 8.1 (3.6) | 246.239 | <0.001 |
| **CNS, n (%)** | 1741 (42.8) | 1265 (28.8) | 221.463 | <0.001 |
| **Cardiovascular, n (%)** | 3366 (82.7) | 3026 (68.9) | 211.256 | <0.001 |
| **Respiratory, n (%)** | 3639 (89.4) | 3443 (78.4) | 181.926 | <0.001 |
| **Hematology, n (%)** | 1686 (41.4) | 1246 (28.4) | 163.009 | <0.001 |
| **Hepatic, n (%)** | 1039 (25.5) | 740 (16.8) | 99.229 | <0.001 |
| **Tachypnea, n (%)** | 3596 (88.4) | 3628 (82.6) | 61.561 | <0.001 |
| **Congestive Heart Failure, n (%)** | 717 (17.6) | 531 (12.1) | 52.733 | <0.001 |
| **Active Cancer, n (%)** | 717 (17.6) | 547 (12.4) | 47.177 | <0.001 |
| **Chronic Liver Disease, n (%)** | 343 (8.4) | 210 (4.8) | 47.163 | <0.001 |
| **Other Chronic Disabling Condition, n (%)** | 1009 (24.8) | 831 (18.9) | 45.779 | <0.001 |
| **Serious Trauma, n (%)** | 252 (6.2) | 449 (10.2) | 44.172 | <0.001 |
| **Infection Source, n (%)** | 1958 (48.1) | 1783 (40.6) | 42.565 | <0.001 |
| **Race: Caucasian, n (%)** | 1761 (43.3) | 2192 (49.9) | 33.223 | <0.001 |
| **AIDS, n (%)** | 56 (1.4) | 10 (0.2) | 27.814 | <0.001 |
| **Tachycardia, n (%)** | 3721 (91.4) | 3879 (88.3) | 27.762 | <0.001 |
| **Days in ICU, Mean (SD)** | 13.2 (15.0) | 15.1 (16.0) | 27.673 | <0.001 |
| **Fungal Infection, n (%)** | 420 (10.3) | 343 (7.8) | 24.200 | <0.001 |
| **Leukocytosis or Leukopenia, n (%)** | 3485 (85.6) | 3611 (82.2) | 24.140 | <0.001 |
| **HIV, n (%)** | 65 (1.6) | 23 (0.5) | 20.991 | <0.001 |
| **Surgical, n (%)** | 1588 (39.0) | 1901 (43.3) | 15.640 | <0.001 |
| **ICU Type: Surgical, n (%)** | 475 (11.7) | 630 (14.3) | 13.168 | <0.001 |
| **Chronic Renal Failure, n (%)** | 482 (11.8) | 417 (9.5) | 12.788 | <0.001 |
| **ICU Transferred, n (%)** | 3773 (92.7) | 4024 (91.6) | 12.303 | <0.001 |
| **Diabetes, n (%)** | 1003 (24.6) | 960 (21.8) | 10.530 | 0.001 |
| **Infection Site: Lungs, n (%)** | 1811 (44.5) | 1859 (42.3) | 5.641 | 0.018 |
| **Chronic Lung Disease, n (%)** | 618 (15.2) | 614 (14.0) | 2.883 | 0.090 |
| **Sex: Male, n (%)** | 2429 (59.7) | 2661 (60.6) | 0.659 | 0.417 |
| **Thermo Dysregulation, n (%)** | 3042 (74.8) | 3284 (74.7) | 0.113 | 0.737 |

P-values are from logistic regression analysis with hospital mortality as response variable.

Total number of OD does not include records with missing OD data.

N= 505, records with mortality status missing are excluded.

OD= Organ Dysfunction, APACHE= Acute Physiology and Chronic Health Evaluation, SD= Standard Deviation, SOFA= Sequential Organ Failure Assessment, CNS= Central Nervous System, AIDS= Acquired Immune Deficiency Syndrome, ICU= Intensive Care Unit, HIV= Human Immunodeficiency Virus.

**Propensity Score and Model Development:**

Propensity scores were derived by constructing multivariate models in which observed patient characteristics were used to predict the probability of receiving a particular treatment. Each patient in the study was assigned a propensity score that represented the estimated probability of receiving a low-dose corticosteroid. Patients were sub-classified into quintiles on the basis of their propensity scores in order to achieve approximate balance in the treatment arms across all measured covariates (see Table S5 in Additional data file 2). These quintile assignments along with treatment and additional factors were used as covariates in the development of a series of logistic regression models with the dichotomous response of Mortality vs. non-Mortality.

The propensity model was initially constructed using logistic regression. Differences between the two treatment groups for individual variables were assessed using Wilcoxon Rank Sum tests for continuous variables and chi-square tests for qualitative variables. Variables with P-values less than 0.10 were declared to be candidate covariates and were included in logistic regression models and were removed using an alpha cut-off of 0.10. Inclusions of interactions between covariates and the propensity model were assessed but not included in any of the models. Covariates used in the final propensity score were age, seven types of ODs, surgical status, chronic lung disease status, active cancer status, and other chronic disabling condition. Significant baseline variables not included as candidates in the propensity score due to missing data were SOFA scores (56% missing data), APACHE II scores (28% missing data) and Fungal Infection (21% missing data). As observed in Table S5 below, application of propensity scores led to an improvement in baseline balance across the two treatment groups in comparison to the imbalances that were present in the raw baseline data.

**ADDITIONAL DATA FILE 2, Table S5: Improvement in Baseline Imbalance Using Propensity Score Analysis**

|  | | **Quintile 1** | | | **Quintile 2** | | | **Quintile 3** | | | **Quintile 4** | | | **Quintile 5** | | |
| --- | --- | --- | --- | --- | --- | --- | --- | --- | --- | --- | --- | --- | --- | --- | --- | --- |
|  | **Original P-value*** | **LDC-No**  **N=1198** | **LDC-Yes**  **N=252** | **p-value** | **LDC-No**  **N=1045** | **LDC-Yes**  **N=406** | **p-value** | **LDC-No**  **N=966** | **LDC-Yes**  **N=485** | **p-value** | **LDC-No**  **N=847** | **LDC-Yes**  **N=604** | **p-value** | **LDC-No**  **N=720** | **LDC-Yes**  **N=731** | **p-value** |
| **Age (mean)** | <0.001 | 52.9 | 54.8 | 0.20 | 56.7 | 57.6 | 0.50 | 61.3 | 60.5 | 0.03 | 63.4 | 64.0 | 0.65 | 68.5 | 68.3 | 0.57 |
| **Organ**  **Dysfunctions (%):**  **Cardiovascular**  **Respiratory**  **Hematology**  **Renal**  **Hepatic**  **Metabolic**  **CNS** | <0.001  <0.001  <0.001  <0.001  <0.001  <0.001  <0.001 | 12.8  65.5  19.4  18.4  13.1  19.4  24.8 | 17.9  65.1  25.0  16.7  9.9  26.2  23.4 | 0.03  0.89  0.05  0.50  0.17  0.01  0.64 | 72.4  69.7  22.1  26.6  15.9  26.2  24.5 | 73.4  69.5  24.4  23.4  16.5  30.3  20.9 | 0.71  0.94  0.35  0.21  0.77  0.12  0.15 | 92.9  86.4  26.8  35.2  16.1  39.5  32.8 | 92.8  87.8  27.2  39.8  15.7  37.1  31.1 | 0.96  0.46  0.87  0.09  0.81  0.37  0.52 | 98.7  94.8  39.1  65.4  23.4  61.2  40.7 | 97.5  95.7  39.2  64.6  25.8  58.9  40.9 | 0.09  0.44  0.95  0.74  0.28  0.40  0.95 | 99.3  98.2  62.4  79.2  33.5  78.2  60.3 | 99.9  98.6  59.2  82.6  34.2  78.7  68.7 | 0.10  0.50  0.22  0.09  0.77  0.83  0.00 |
| **Surgical Status (%)** | <0.001 | 33.3 | 27.0 | 0.05 | 37.6 | 34.2 | 0.23 | 42.2 | 40.4 | 0.51 | 40.4 | 43.0 | 0.31 | 46.7 | 54.0 | 0.01 |
| **Chronic Lung Disease (%)** | <0.001 | 3.5 | 5.2 | 0.21 | 9.7 | 12.6 | 0.11 | 8.8 | 7.8 | 0.53 | 12.3 | 14.7 | 0.17 | 38.5 | 38.7 | 0.92 |
| **Active Cancer (%)** | <0.001 | 7.3 | 7.5 | 0.88 | 11.5 | 11.3 | 0.93 | 14.3 | 15.9 | 0.42 | 19.6 | 19.5 | 0.98 | 28.3 | 29.7 | 0.57 |
| **Other Chronic Disabling Condition (%)** | <0.001 | 11.9 | 11.9 | 0.99 | 15.9 | 13.5 | 0.27 | 20.4 | 18.4 | 0.36 | 20.2 | 21.9 | 0.44 | 35.1 | 40.8 | 0.03 |

* Excerpted from Table 2

LDC= Low-Dose Corticosteroids, CNS= Central Nervous System
